# Hepcidin (rs10421768), Transferrin (rs3811647, rs1049296) and Transferrin Receptor 2 (rs7385804) Gene Polymorphism Might Be Associated with the Origin of Multiple Sclerosis

**DOI:** 10.3390/ijerph19116875

**Published:** 2022-06-04

**Authors:** Laura Stachowska, Dorota Koziarska, Beata Karakiewicz, Artur Kotwas, Anna Knyszyńska, Marcin Folwarski, Karolina Dec, Ewa Stachowska, Viktoria Hawryłkowicz, Monika Kulaszyńska, Joanna Sołek-Pastuszka, Karolina Skonieczna-Żydecka

**Affiliations:** 1 Department of Human Nutrition and Metabolomics, Pomeranian Medical University in Szczecin, Broniewskiego 24, 71-460 Szczecin, Poland; laura.stachowska@pum.edu.pl (L.S.); karolinatdec@gmail.com (K.D.); ewa.stachowska@pum.edu.pl (E.S.); vik.hawrylkowicz@gmail.com (V.H.); 2 Department of Neurology, Pomeranian Medical University in Szczecin, Unii Lubelskiej 1, 72-252 Szczecin, Poland; dorota.koziarska@pum.edu.pl; 3 Subdepartment of Social Medicine and Public Health Department of Social Medicine, Pomeranian Medical University in Szczecin, Żołnierska 48, 71-210 Szczecin, Poland; beata.karakiewicz@pum.edu.pl (B.K.); artur.kotwas@pum.edu.pl (A.K.); 4 Department of Functional Diagnostics and Physical Medicine, Pomeranian Medical University in Szczecin, 71-210 Szczecin, Poland; anna.knyszynska@pum.edu.pl; 5 Department of Clinical Nutrition and Dietetics, Medical University of Gdansk, 80-211 Gdańsk, Poland; marcin.folwarski@gumed.edu.pl; 6 Department of Biochemical Science, Pomeranian Medical University in Szczecin, Broniewskiego 24, 71-460 Szczecin, Poland; monika.kulaszynska@pum.edu.pl; 7 Department of Anaesthesiology and Intensive Therapy, Pomeranian Medical University in Szczecin, Unii Lubelskiej 1, 72-252 Szczecin, Poland; joanna.solek.pastuszka@pum.edu.pl

**Keywords:** multiple sclerosis, neurodegeneration, iron, polymorphism

## Abstract

Multiple sclerosis (MS) is a demyelinating disease of the central nervous system in which there is a multifocal damage to the nerve tissue. Additionally, the literature emphasizes the excessive accumulation of iron in the central nervous system of patients, which is negatively correlated with their psychophysical fitness. Iron metabolism genes polymorphisms may modulate iron deposition in the body and thus affect the clinical course of MS. We aimed to assess the frequency of *HAMP*, *TFR*2, and *TF* polymorphisms in MS patients and their impact on the clinical course of the disease. The studied polymorphisms were identified by the Real-Time PCR using TaqMan technology. Neurological assessment by means of EDSS scale was conducted. This cross-sectional study included 176 patients, with the mean age of onset of symptoms at 30.6 years. The frequency of alleles of the studied polymorphisms was as follows: (a) *HAMP* rs10421768: A 75.9% (*n* = 267), G 24.1% (*n* = 65), (b) *TF* rs1049296: C 89.2% (*n* = 314), T 10.8% (*n* = 38), (c) *TF* rs3811647: A 39.8% (*n* = 140), G 60.2% (*n* = 212), (d) *TFR*2 rs7385804: A 59.1% (*n* = 59.1%), C 40.9% (*n* = 144). In the codominant inheritance model of *TF* rs1049269, it was shown that people with the CT genotype scored statistically significantly lower points in the EDSS scale at the time of diagnosis than those with the CC genotype (CC Me = 1.5, CT Me = 1.0 *p* = 0.0236). In the recessive model of *TF* inheritance rs3811647, it was noticed that the primary relapses were significantly more frequent in patients with at least one G allele compared with those with the AA genotype (AG + GG = 81.2%, AA = 18.8%, *p* = 0.0354). In the overdominant model rs7385804 *TFR*2, it was shown that among patients with the AA genotype, multiple sclerosis occurs significantly more often in relatives in a straight line compared with people with the AC and CC genotypes (AA = 100.0%, AC + CC = 0.0%, *p* = 0.0437). We concluded that the studied polymorphisms might affect the clinical course of MS.

## 1. Introduction

Multiple sclerosis (MS) is an inflammatory disease of the CNS in which demyelination and axonal damage followed by patient death occur. Exact etiology of these disorders has not been established yet. It is commonly accepted that MS is an autoimmune disease, and CD4 + T cells being responsible for the synthesis of interferon and interleukin 17 play a significant role in its pathogenesis. Both processes are closely related to the iron balance in a human body. In the histopathological and magnetic resonance imaging examinations a disruption in iron accumulation in the areas of gray and white matter within the brain at very early stages of the disease was observed [1]. It has been presumed that the intensity of iron deposition may be correlated with the duration or severity of the disease [1,2].

Haider distinguished three key mechanisms in the formation of neurotoxic reactive oxygen species, namely: i. Stimulation of free radicals by inflammation involving immune cells, ii. excess iron release during demyelination, and iii. disturbances in energy metabolism due to mitochondria damage [3]. Iron plays a role in each of these mechanisms. It is assumed that the iron dose delivered to the intercellular space as a result of the breakdown of the myelin sheath constitutes the first explosion of oxidative stress [4]. Its accumulation in the microglia structures may stimulate inflammation and, consequently, lead to a self-propelling feedback loop [5].

Taking into account the biological demand for iron and its predisposition to toxicity, it is necessary to maintain the homeostasis of this biometal very precisely.

### 1.1. Transport and Absorption of Iron in the Brain

Iron cannot freely pass from the bloodstream to the brain. Its absorption occurs through prior binding to transferrin (*TF*) and crossing the blood-brain barrier (BBB) or the cerebrospinal fluid (CSF), which controls its flow, preventing possible iron overload, with vascular endothelial cells (BVCEs) mainly coordinating the BBB action [6,7,8].

*TF* is a protein carrier that binds two iron atoms (Fe^3+^) with high affinity. The formation of the *TF*–Fe2 complex is pH dependent and occurs best in a neutral environment [9]. *TF* plays a key role in the distribution and maintenance of iron homeostasis. It intermediates between the places of its storage, absorption, and use, delivering to all cells, including BVECs [10]. The hydrophilic nature of holotransferin (iron-binding *TF*) prevents it from penetrating the brain, so the iron bound to transferrin (*TF*–Fe2) can be incorporated into the BVCEs only due to the presence of transferrin receptors 1 and 2 (*TFR*1, *TFR*2) on their surface [11,12]. BVECs express around 100,000 cellular receptors on the cell membrane and are the primary iron uptake pathway, allowing the *TF*–Fe2 complex to penetrate through the endocytic process [7,13].

*TFR*1 as the main receptor for *TF* is abundant throughout the nervous system, especially in neurons [14]. *TFR*2 has a lower affinity for *TF* than *TFR*1, it is mainly expressed in the mitochondria of dopaminergic neurons and, unlike *TFR*1, it is not controlled by intracellular iron levels because it lacks iron-sensitive elements [14,15].

Due to the important role of transferrin and its receptors in iron transport, these structures are still the subject of extensive research. Rs1049296 *TF* is the basis for typing C1 (*TF*C1)/C2 (*TF*C2) transferrin. The C allele encodes the C1 subtype, while the T-allele, the less common one, is responsible for the C2 subtype, which results from the conversion of proline to serine (Pro570Ser mutation) at the 570 C-terminal site of native *TF*C1 [16]. The association study provided evidence that rs1049296 on chromosome 3 influences the glycosylation of transferrin, changing its structure [17]. The rs3811647 *TF* polymorphism seems to be of equal importance. There are studies showing its significant relationship with *TF* concentrations among Europeans [18], and McLaren et al. documented its association with iron deficiency in the American population [19]. Blanco-Rojo et al., demonstrated in vitro that the A allele more strongly induces transferrin expression compared with the G allele and hypothesized that it may serve as a binding site for the glucocorticoid receptor (GR) [20]. Research indicates the participation of *TFR*2 in the regulation of iron levels by influencing the indirect activation of hepcidin, the main regulator of iron levels in the body [21,22]. Pichler et al., indicated the *TFR*2 gene as a possible variant regulating iron levels in clinically healthy people, while proving the relationship of rs7385804 with the levels of *TFR*2 mRNA expression in the human liver [21].

### 1.2. Regulation of Iron Metabolism—The Role of Hepcidin

Iron metabolism in the CNS is coordinated by two regulatory systems. The first one controls iron metabolism at the cellular level through the post-transcriptional regulation of iron regulating proteins, and the second one acts at the systemic level by the use of hepcidin, a hormone regulating the expression of ferroportin (FPN) [22].

Hepcidin (HAMP) is a protein hormone that is mainly expressed in hepatocytes, but recent research demonstrated the presence of hepcidin in the brain, pancreas, and heart. The mechanism of iron regulation in the cell by HAMP is mainly based on the control of FPN expression at the level of its translation [6]. Hepcidin in response to excessive iron levels binds FPN and phosphorylates its tyrosine residues, which in turn leads to its lysosomal degradation [22]. FPN is the only exporter of iron, so the inhibition of its synthesis results in the complete accumulation of this element. The opposite effect can be seen in the case of iron deficiency. It has been documented that in rats with iron deficiency there was a strong decrease in the *HAMP* expression [23,24]. Current studies report not only the inhibitory effect of hepcidin on export, but also on the import of iron via Divalent Metal (Ion) Transporter 1 (DMT1) and Transferrin receptor protein 1 (TFR1) [23]. It is assumed that this may be related to the hitherto unknown HAMP receptor on the astrocyte membrane, which activates AMP-activated kinase (AMPK) intracellularly [25]. Unfortunately, detailed information on the signaling pathways for the stimulation and inhibition of HAMP synthesis in response to any changes in iron levels is currently limited.

One of the most frequently studied polymorphisms of the *HAMP* gene is rs10421768 A > G. It has been hypothesized that the presence of the G allele promotes reduction of hepcidin transcription, thus enhancing iron absorption [26]. Parajes et al., in turn, showed that the c.-582G variant slightly reduced the transcriptional activity of the *HAMP* promoter in vitro, because it was located in the E-box, which is placed in the area responsive to transcription factors and could lead to a slight reduction in the synthesis of this protein, although this did not significantly influence the concentration of iron in the blood serum [27]. Liang et al. observed a reduced hepcidin production by CD14 + monocytes among people belonging to the Chinese population carrying the GG genotype compared with cells of people carrying at least one A allele in the genotype [28].

According to the literature data, excessive iron accumulation is observed in the central nervous system of patients suffering from MS, which is negatively correlated with their psychophysical fitness. The causes of this phenomenon are not fully understood. The presence of polymorphisms in the genes of iron metabolism may modulate iron deposition in the body and thus affect the clinical course of the disease. In the light of these data, it seems justified to investigate the frequency of the rs10421768 A > G polymorphism in the *HAMP* gene, rs7385804 A > C *TFR*2 and rs1049296 C > T, and rs3811647 G > A in *TF* in MS patients and to try to link the genotype with the clinical phenotype of the disease.

## 2. Materials and Methods

### 2.1. Study Group

This cross-sectional study included 176 patients (55 males and 121 females) with a clinical diagnosis of MS, under the care of the Provincial Center for Demyelinating Diseases in Szczecin. Patients’ disability was assessed by two neurologists at diagnosis and in 2019 using Kurtzki’s Expanded Disability Status Scale (EDSS) [14].

Additionally, based on the clinical interview and collected data charts, we retrieved information on:

- EDSS rating when patient was diagnosed with MS,
- the age of onset,
- the co-occurrence of other autoimmune disorders,
- the occurrence of autoimmune disorders in family history,
- the cases of MS among relatives,
- de novo diagnosis of MS,
- the presence of relapses,
- the number of affected systems,
- type of the disease,
- disease duration time.

All study participants signed an informed consent form. The study received a positive opinion of the Bioethics Committee at the Pomeranian Medical University in Szczecin (Consent number KB-0012/163/12).

### 2.2. Molecular Research Methodology

#### 2.2.1. DNA Isolation

Genomic DNA was isolated from patients’ peripheral blood leukocytes using the ExtractMe DNA Blood Kit (BLIRT, Gdańsk, Poland), according to the manufacturer’s instructions. The DNA isolates were stored at −20 °C until analysis.

#### 2.2.2. Identification of the Studied Polymorphisms

Genotyping was performed by real-time polymerase chain reaction (PCR) using a Cycler^®^ 96 System lamp (Roche Diagnostics, Pleasanton, CA, USA) and TaqMan probes (Life Technologies, Foster City, CA, USA). The excited signals were detected with FAM and VIC fluorescent dyes. *HAMP* rs10421768, *TFR2* rs7385804, *TF* rs1049269, and *TF* rs3811647 test identifiers were C___2604942_10, C___2184545_10, C___7505275_10, and C__27492858_10 respectively.

The reaction mixture (10 μL) contained:

- 1 μL of genomic DNA,
- 5 μL of Taq Man Genotiping Master Mix (Life Technologies, Foster City, CA, USA),
- 3.75 μL PCR Grade Water (Life Technologies, Foster City, CA, USA),
- 0.25 μL TaqMan probe (Life Technologies, Foster City, CA, USA).

Real Time PCR was performed under the following conditions:

- Pre-incubation (1 cycle): 300 s—95 °C,
- 2-stage Amplification (50 cycles):
  1. 95 °C × 15 s
  2. 60 °C × 60 s.

The cooling step was omitted.

### 2.3. Satistical Analysis

Statistical analysis was performed using the MedCalc software ver. 19.2 (Ostend, Belgium). The distribution of continuous variables was different from normality; therefore, the data were presented as medians and quartile ranges. Courts’ online calculator (2005–2008) was used to determine compliance with the Hardy–Weinberg equilibrium. Statistical inference was based on the Mann–Whitney U test or the Kruskal–Wallis test, as appropriate. Chi-square/Fisher’s exact approach was used for qualitative data. The level of significance was set as *p* < 0.05, while *p* = 0.05–0.1 was considered as the area of the statistical trend.

The following genetic inheritance models for *HAMP*, *TF,* and *TFR2* were analyzed in order to check their impact on the course of the disease and the rate of progression in MS patients:

- Over dominant (heterozygous vs. homozygous recessive + homozygous dominant)
- Dominant (dominant homozygous vs. heterozygous + recessive homozygous
- Recessive (homozygous recessive vs. heterozygous + dominant homozygous)
- Codominant (recessive homozygous vs. heterozygous vs. dominant homozygous)

## 3. Results

### 3.1. Characteristics of the Study Group

The study group consisted of 68.75% females (*n* = 121) and 31.25% males (*n* = 55). The EDSS score, both at the time of diagnosis and at the time of this study (2019) was not sex dependent, nor was the age of onset (*p* > 0.05) (Table 1). De novo MS was found in 61.9% (*n* = 109) of patients. Additionally, 41.5% (*n* = 73) of patients had a single-focal onset and 58.5% (*n* = 103) had multifocal onset of the MS. Primary projections were diagnosed in 36.4% (*n* = 64) of patients.

MS occurred in the study group in the following forms:

- In 97.2% (*n* = 171) relapsing–remitting multiple sclerosis (RR),
- In 2.3% (*n* = 4) secondary progressive multiple sclerosis (SP),
- In 0.6% (*n* = 1) primary progressive multiple sclerosis (PP).

The mentioned clinical parameters were also analyzed for differences in sex categories. A significantly higher prevalence of autoimmune diseases was observed among females compared with the opposite sex (*n* = 12 vs. *n* = 0, *p* = 0.016). In addition, the presence of MS in the lateral line was observed significantly more frequently among females compared with males (*n* = 11 vs. *n* = 0, *p* = 0.02).

There was a tendency for the higher incidence of MS in the family in females compared with males (*n* = 15 vs. *n* = 2, *p =* 0.07). Only females were found to have other autoimmune diseases compared with the opposite sex. Moreover, only in females was the presence of collateral MS confirmed. Additional data are included in Table 2.

### 3.2. Genotyping

The genotype distributions and allele frequencies of *HAMP* rs10421768 A > G, *TF* rs1049296 C > T, *TF* rs3811647 G > A, *TFR2* rs7385804 A > C polymorphisms are shown in Table 3. Deviations from Hardy-Weinberg Law were observed only for *TF* rs3811647 G > A polymorphism (X^2^ = 4.6, *p* = 0.03), in the remaining cases (polymorphisms *TF* rs1049296 C > T (X^2^ = 2.6, *p* = 0.11), *HAMP* rs10421768 A > G (X^2^ = 0.0, *p* = 0.91) and *TFR2* rs7385804 A > C (X^2^ = 0.2, *p* = 0.65) were consistent.

Additionally, there was a tendency in the dominant model for *TF* rs3811647 to the presence of a higher number of points in the EDSS scale at the time of diagnosis for persons with the GG genotype (GG Max = 6.5, AG + AA Max = 6.0, *p =* 0.0915).

The relationship between the occurrence of *HAMP* polymorphisms rs10421768 A > G, *TF* rs1049296 C > T, *TF* rs3811647 G > A, and *TFR*2 rs7385804 A > C was examined with respect to the number of points in the EDSS scale at the diagnosis of the disease, the EDSS assessment in 2019, and the age of the first symptoms. Data are presented in Table 4, Table 5, Table 6, Table 7 and Table 8. No statistically significant correlation was found between the *HAMP* rs10421768, *TFR*2 rs7385804, and *TF* rs3811647 genotypes and the subjects with continuous variables in any of the analyzed inheritance models. In the case of the rs1049269 codominant model of the *TF* polymorphism, the analysis showed that people with the CT genotype scored statistically significantly lower points in the EDSS scale at the diagnosis of the disease than those with the CC genotype (CC Me = 1.5, CT Me = 1.0 *p =* 0.0236). The remaining variables for rs1049269 were not statistically significant related to the parameters tested in any of the analyzed inheritance models. The data are presented in Table 8.

Then, the relationship between the studied polymorphisms and selected clinical parameters was assessed. Data are presented in Table 8, Table 9, Table 10 and Table 11. The relationships between the analyzed qualitative variables in each of the tested inheritance models of *HAMP* rs10421768 and *TF* rs1049269 polymorphisms do not exist. However, it was observed that primary relapses were significantly more frequent in patients with AG and GG genotypes compared with the AA genotype in the recessive inheritance model for *TF* rs3811647 (AG + GG = 81.2%, AA = 18.8%, *p =* 0.0354). A statistical trend has been shown regarding the tendency to occur primary relapses more frequently among persons with the AA + AG genotype compared with GG patients (GG = 25.0%, AA + AC = 75.0%, *p =* 0.0923) in the recessive *HAMP* rs10421768 inheritance model.

## 4. Discussion

Iron plays a key role in human physiology. It takes part, among others, in the transport and storage of oxygen, but is also acts as a cofactor of basic metabolism enzymes, antioxidant enzymes, and participates as a prosthetic group in many key processes, such as myelination and remyelination of axons. Due to the remarkable ability to change the redox potential, skewed iron homeostasis may result in serious neuronal disorders, the most toxic of which is the formation of reactive oxygen species (ROS). ROS can enhance damage to the mitochondrial proteins of the iron–sulfur cluster in the respiratory chain, while inducing uncontrolled release of this element, magnifying further damage [29]. The CNS is very susceptible to any oxidative damage due to the low activity of the enzymes responsible for ROS removal, such as catalase, superoxide dismutase, or glutathione peroxidase [29]. Increased oxidative stress can lead to the death of neurons, resulting in neurodegeneration. Excessive iron accumulation in brain cells has been observed in patients suffering from neurodegenerative diseases such as MS, Parkinson’s Disease, and Alzheimer’s Disease.

MS is a chronic disease of the CNS, leading to oligodendrocyte damage, demyelination, and astrocytic scar formation [4]. Choi et al., documented decreased levels of glutathione in the brains of patients with secondary progressive form compared with a control group of healthy people, confirming the increased susceptibility to oxidative stress in MS patients [30]. Mahad et al., in their work, listed the consequences of mitochondrial changes in the course of MS [31]. The first was the energy deficit, which may result in functional disorders, while in a more severe course, it was associated with structural damage with permanent consequences, such as damage to the nervous tissue due to axonal degeneration [31]. Mitochondrial damage can also result in an electron release cascade that drives ROS formation, leading to a self-propelling loop based on positive feedback. This is a vicious cycle that leads to tissue wasting.

It was shown that iron metabolism in the brain is disturbed in the course of MS, but little is known about the genetic basis of this process. There are several proteins responsible for the transport and metabolism of iron that could be potential factors responsible for the dyshomeostasis of this element in the brain. Based on the current research, the aim of this study was to determine the effect of polymorphisms of key genes for iron homeostasis on the occurrence and development of MS in patients from the area of West Pomerania in Poland. Based on the literature data, the *HAMP* rs10421768 A > G, *TFR*2 rs7385804 A > C, *TF* rs3811647 G > A, and rs1049296 C > T were selected for the analysis [32,33,34].

In order to determine the impact of the studied polymorphism on the incidence and disease progression, an analysis was carried out in a group of 176 patients under the care of the Neurology Clinic in Szczecin. The present study showed an association between the carrier of 1 mutant rs1049269 *TF* polymorphism allele in the codominant model and a lower EDSS score obtained at diagnosis. This means that the heterozygous allelic system present in this model may have some protective character, the exponent of which is the number of EDSS points at the diagnosis of the disease. The obtained analyses also showed that the lower EDSS score calculated in the case of diagnosis is correlated with slower and milder progression of changes in the course of MS, as evidenced by the lower number of points in the EDSS scale obtained again in 2019.

With regard to the rs3811647 of the *TF* gene, there was a tendency to have a higher number of points in the initial EDSS scale for people with the GG genotype. This result, although not statistically significant in any way, makes it possible to suspect a relationship between the G allele and the presence of more severe neurological symptoms at diagnosis. On the other hand, at least one C allele in the single-nucleotide polymorphism (SNP) rs7385804 *TFR*2 range may be associated with de novo diagnosis of the disease, because patients with AC and CC genotypes are significantly less likely to develop MS in straight line relatives. At a later stage, a significantly more frequent occurrence of primary relapses was observed in patients with the AG and GG genotypes than in those with the AA genotype in the range of *TF* rs3811647, which means that the presence of at least one genotype A has a protective effect on this clinical parameter. However, in terms of the *HAMP* rs10421768 polymorphism in the recessive model, a tendency to a higher incidence of primary relapses was documented among people with the AA + AG genotype compared with patients with the GG genotype. Perhaps this result, after conducting the analysis on a larger study group, could turn out to be statistically significant and suggest the aggravating nature of the A allele of this SNP for patients with MS. No other relationships between other clinical parameters and *HAMP* rs10421768, *TFR*2 rs7385804, *TF* rs1049269, and *TF* rs3811647 genotypes were observed in any of the analyzed inheritance models.

With regard to the rs3811647 of the *TF* gene, there was a tendency to have a higher number of points in the initial EDSS scale for people with the GG genotype. This result, although not statistically significant in any way, makes it possible to suspect a relationship between the G allele and the presence of more severe neurological symptoms at diagnosis. On the other hand, at least one C allele in the SNP rs7385804 *TFR*2 range may be associated with de novo diagnosis of the disease, because patients with AC and CC genotypes are significantly less likely to develop MS in straight line relatives. At a later stage, a significantly more frequent occurrence of primary relapses was observed in patients with the AG and GG genotypes than in those with the AA genotype in the range of *TF* rs3811647, which means that the presence of at least one genotype A has a protective effect on this clinical parameter. However, in terms of the *HAMP* rs10421768 polymorphism in the recessive model, a tendency toward a higher incidence of primary relapses was documented among people with the AA + AG genotype compared with patients with the GG genotype. Perhaps this result, after conducting the analysis on a larger study group, could turn out to be statistically significant and suggest the aggravating nature of the A allele of this SNP for patients with MS. No other relationships between other clinical parameters and *HAMP* rs10421768, *TFR*2 rs7385804, *TF* rs1049269, and *TF* rs3811647 genotypes were observed in any of the analyzed inheritance models.

The molecular mechanism of iron metabolism disorders in MS remains unclear, but the available literature data indicate its multifactorial nature with the participation of genetic variants. Presumably, the process of harmful accumulation of intracellular iron, along with its systemic deficiency, could occur as a consequence of overexpression of hepcidin, which reduces FPN on the cell surface. This hypothesis is confirmed by the fact that its expression in neurons and astrocytes depends on microglia, which in the course of MS maintain inflammation [35,36,37]. It is worth mentioning that factors such as tumor necrosis factor (TNF-alpha) or interleukin 6 (IL-6) are inducers of hepcidin expression in nervous tissue [6]. In addition, it has been confirmed that in response to the synthesis of pro-inflammatory factors by microglia, not only the increased synthesis of hepcidin and, consequently, the degradation of ferroportin with iron accumulation occur, but also the expression of DMT1, responsible for its import into the cell, is increased [35,38].

The transport of iron in the brain area is mainly in the form associated with *TF* or to a small extent as non-*TF* iron (NTBI) [36], hence the authors’ interest in two variants of the *TF* gene. Bartzokis et al., suggested in their study that men carrying the H63D variant of the HFE gene and the *TF*C2 variant (TT for rs1049296 *TF*) may have a higher risk of developing Alzheimer’s disease [39]. Wang confirmed this in a meta-analysis on rs1049296 *TF*C2, which turned out to be an essential determinant of the risk of AD [40]. Kutalik et al,. provided evidence that this polymorphism influences the glycosylation of transferrin contributing to the overall genetic effect [17].

The rs3811647 *TF* polymorphism is also widely analyzed. The genome-wide association study conducted by de Tayrac et al., was aimed at determining its role as a factor modifying iron metabolism in the course of haemochromatosis. SNP rs3811647 turned out to be clearly related to the serum level in the European population [18]. Peng An et al., documented the association between rs3811647 *TF* and decreased serum transferrin concentrations and total iron binding capacity (TIBC) and SNP *TFR*2 rs7385804 and lower serum iron levels (SI) [34]. It should be emphasized, however, that both genetic variants were not classified as significant for the prevalence of anemia in elderly women in the Chinese population [34]. Pichler et al., in 2011, for the first time demonstrated that *TFR*2 has an effect on iron levels in people without obvious clinical symptoms [21]. Additionally, mutations in the *TFR*2 gene lead to the occurrence of type 3 haemochromatosis in humans [41]. This polymorphism is involved in the control of iron levels, possibly by influencing the activation of hepcidin expression [21].

It should be mentioned that although the literature on the subject is not extensive, the results of own research are not consistent with those presented by other researchers. For example, Andreani et al., showed that SNP rs10421768 is probably related to the *HAMP* promoter functions and the A > G substitution may predispose to overload iron levels in patients with thalassemia [33]. This hypothesis was confirmed by Zarghamian et al., in 2020, showing that the GG genotype was associated with an overload of iron levels in the heart of patients with β-thalassemia not responding to iron chelation therapy [42]. This is another confirmation of the hypothesis that when A > G nucleotide is substituted, there is a significant reduction in *HAMP* gene transcription due to impeded attachment of transcription factors from the E-Box in the promoter area. However, different results were obtained by Parajes, who performed an analysis of the influence of genetic variants on *HAMP* expression in vitro, ruling that the A > G variant only led to a subtle reduction in its expression and found no significant relationship between the presence of GG homozygote and iron concentration, and transferrin levels in serum and its saturation in the Galician population [27]. To add, it should be taken into account that in vitro conditions are not able to fully reflect the in vivo conditions.

There are many hypotheses about the role of iron in the development of MS, and the question remains unresolved. At present, it is still unclear whether iron overload of neurons is a primary mechanism accompanying multiple sclerosis or a consequence of its occurrence. The first scenario is the fact that the toxic accumulation of iron in the brain may affect the development of the disease by activating microglia and stimulating this structure to produce pro-inflammatory cytokines [26]. The iron-overloaded microglia is deprived of the ability to actively remove myelin residues, which prevents proper remyelination processes [43,44]. Additionally, this hypothesis is supported by data on oxidative stress and its influence on apoptosis of nerve cells. On the other hand, there are reports of the mitigating effect of iron on demyelinating changes. Lee et al., in their study on an experimental autoimmune model of encephalomyelitis (EAE) induced in a marmoset (a species of small monkey), concluded that iron is not associated with early inflammation [43]. Moreover, his results suggested that iron accumulation contributes to the repair of demyelinating lesions rather than its spontaneous induction [43].

## 5. Conclusions

The results of this study showed a significantly lower degree of neurological disability of the patient at the time of diagnosis related to rs1049269 *TF* heterozygotes, lower incidence of primary relapses in patients with genotype AA in the recessive model of inheritance regarding *TF* rs3811647, and a possible association of the C allele of the rs7385804 *TFR*2 SNP with de novo diagnosis of the disease. In order to obtain more accurate results, the relationship of certain genetic variants with the hematological parameters of patients should be investigated. The lack of analyses of iron concentrations in the blood serum of the examined people is the greatest limitation of the presented study. An analysis of the genotype–phenotype relationship would better document the role of iron management in the pathogenesis/course of MS.

## Figures and Tables

**Table 1:** Study group characteristics.

Clinical Parameters	Sex												*p*
	Females						Males						
	*n*	Min	Max	M	Me	25–75 P	*n*	Min	Max	M	Me	25–75 P	
EDSS 2019 (points)	121	0.0	6.5	2.0	1.5	1.0–2.0	55	0.0	6.0	2.3	1.5	1.0–3.5	0.28
EDSS at time of diagnosis (points)	121	0.0	6.5	1.7	1.5	1.0–2.0	55	0.0	6.0	1.9	1.5	1.0–2.9	0.19
Age at clinical onset (years)	121	15.0	62.0	30.6	29.0	24.0–37.3	55	16.0	64.0	29.3	27.0	21.0–33.8	0.21

*n*—number of individuals, Min—minimum, Max—maximum, 25–75 P—interquartile ranges, M—mean, Me—median, *p*—statistical significance.

**Table 2:** Clinical parameters by sex.

Parameter		Sex		*n* (%)	X^2^	*p*
		Females	Males			
		*n*	*n*			
Autoimmune diseases Presence	No	109	55	164 (93.2%)	5.8	0.016
	Yes	12	0	12 (6.8%)		
	Overall	121 (68.7%)	55 (31.2%)	176 (100%)		
Family autoimmune diseases history	No	89	39	128 (72.7%)	0.1	0.716
	Yes	32	16	48 (27.3%)		
	Overall	128 (72.7%)	48 (27.3%)	176 (100%)		
De novo MS phenotype	No	43	24	67 (38.1%)	1.0	0.306
	Yes	78	31	109 (61.9%)		
	Overall	121 (68.7%)	55 (31.2%)	176 (100%)		
Primary projections	No	76	36	112 (63.6%)	0.1	0.736
	Yes	45	19	64 (36.4%)		
	Overall	121 (68.7%)	55 (31.2%)	176 (100%)		
Family history of MS	No	106	53	159 (90.3%)	3.3	0.069
	Yes	15	2	17 (9.7%)		
	Overall	121 (68.7%)	55 (31.2%)	176 (100%)		
Number of occupied systems	One	51	22	73 (41.5%)	0.1	0.932
	Two	47	23	70 (39.8%)		
	Three	23	10	33 (18.8%)		
	Overall	121 (68.7%)	55 (31.2%)	176 (100%)		
MS onset	SF	51	22	73 (41.5%)	0.1	0.789
	MF	70	33	103 (58.5%)		
	Overall	121 (68.7%)	55 (31.2%)	176 (100%)		
MS disease course	PP	0	1	1 (0.6%)	2.3	0.320
	RR	118	53	171 (97.2%)		
	SP	3	1	4 (2.3%)		
	Overall	121 (68.7%)	55 (31.2%)	00%)		
MS in side-line	No	110	55	165 (93.7%)	5.3	0.021
	Yes	11	0	11 (6.2%)		
	Overall	121 (68.7%)	55 (31.2%)	176 (100%)		
MS in straight line	No	118	54	172 (97.7%)	0.1	0.786
	Yes	3	1	4 (2.3%)		
	Overall	121 (68.7%)	55 (31.2%)	176 (100%)		

MS—multiple sclerosis, SF—single focal, MF—multi focal, PP—primary progressive multiple sclerosis, RR—relapsing—remitting multiple sclerosis, SP—secondary progressive multiple sclerosis, *n*—number of individuals, X^2^—Chi-square, *p*—statistical significance.

**Table 3:** Distribution of genotypes in the studied inheritance models among the study group.

SNP	Model of Inheritance	Genotype	*n*	%
*HAMP* rs10421768	Codominant	AA	101	57.4%
		AG	65	36.9%
		GG	10	5.7%
	Dominant	AA	101	57.4%
		AG + GG	75	42.6%
	Overdominant	AG	65	36.9%
		GG + AA	111	63.1%
	Recessive	AG + AA	166	94.3%
		GG	10	5.7%
*TF* rs3811647	Codominant	AA	21	11.9%
		AG	98	55.7%
		GG	57	32.4%
	Dominant	AG + AA	119	67.6%
		GG	57	32.4%
	Overdominant	AA + GG	78	44.3%
		AG	98	55.7%
	Recessive	AA	21	11.9%
		AG + GG	155	88.1%
*TF* rs1049269	Codominant	CC	138	78.4%
		CT	38	21.6%
*TFR2* rs7385804	Codominant	AA	60	34.1%
		AC	88	50.0%
		CC	28	15.9%
	Dominant	AA	60	34.1%
		AC + CC	116	65.9%
	Overdominant	AA + CC	88	50.0%
		AC	88	50.0%
	Recessive	AA + AC	148	84.1%
		CC	28	15.9%

*n*—number of individuals, SNP—Single-nucleotide polymorphism.

**Table 4:** Analysis of the relationship between the rs10421768 *HAMP* polymorphism and selected clinical parameters.

*HAMP* rs10421768						
EDSS 2019 (Points)						
Model of Inheritance	Genotype	*n*	25–75 P	Me	Max	*p*
Codominant	AA	101	1.0–2.5	1.5	6.5	0.397452
	AG	65	1.5–2.3	1.5	6.5	
	GG	10	1.0–3.0	1.5	4.5	
Dominant	AA	101	1.0–2.5	1.5	6.5	0.1836
	AG + GG	75	1.5–3.0	1.5	6.5	
Overdominant	AG	65	1.5–2.3	1.5	6.5	0.1906
	GG + AA	111	1.0–2.9	1.5	6.5	
Recessive	GG	10	1.0–3.0	1.5	4.5	0.9108
	AG + AA	166	1.0–2.0	1.5	6.5	
EDSS at diagnosis (points)						
Model of inheritance	Genotype	*n*	25–75 P	Me	Max	*p*
Codominant	AA	101	1.0–2.0	1.5	5.5	0.526175
	AG	65	1.0–2.0	1.5	6.5	
	GG	10	1.0–3.0	1.5	4.0	
Dominant	AA	101	1.0–2.0	1.5	5.5	0.2706
	AG + GG	75	1.0–2.0	1.5	6.5	
Overdominant	AG	65	1.0–2.0	1.5	6.5	0.3919
	GG + AA	111	1.0–2.0	1.5	5.5	
Recessive	GG	10	1.0–3.0	1.5	4.0	0.5697
	AG + AA	166	1.0–2.0	1.5	6.5	
Age at clinical onset (years)						
Model of inheritance	Genotype	*n*	25–75 P	Me	Max	*p*
Codominant	AA	101	23.0–35.3	30.0	64.0	0.848654
	AG	65	22.0–36.0	28.0	62.0	
	GG	10	24.0–34.0	28.5	42.0	
Dominant	AA	101	23.0–35.3	30.0	64.0	0.6448
	AG + GG	75	25.0–31.0	28.0	62.0	
Overdominant	AG	65	22.0–36.0	28.0	62.0	0.8002
	GG + AA	111	23.3–35.0	29.0	64.0	
Recessive	GG	10	24.0–34.0	28.5	42.0	0.6475
	AG + AA	166	23.0–36.0	29.0	64.0	

*n*—number of individuals, 25–75 P—interquartile ranges, Me—median, Max—maximum, *p*—statistical significance.

**Table 5:** Analysis of the relationship between the rs3811647 *TF* polymorphism and selected clinical parameters.

*TF* rs3811647						
EDSS 2019 (Points)						
Model of Inheritance	Genotype	*n*	25–75 P	Me	Max	*p*
Codominant	AA	21	1.0–2.3	1.5	6.5	0.899106
	AG	98	1.0–3.0	1.5	6.0	
	GG	57	1.0–2.5	1.5	6.5	
Dominant	GG	57	1.0–2.5	1.5	6.5	0.8106
	AG + AA	119	1.0–3.0	1.5	6.5	
Overdominant	AG	98	1.0–3.0	1.5	6.0	0.6639
	AA + GG	78	1.0–2.5	1.5	6.5	
Recessive	AA	21	1.0–2.3	1.5	6.5	0.7489
	AG + GG	155	1.0–2.8	1.5	6.5	
EDSS at diagnosis (points)						
Model of inheritance	Genotype	*n*	25–75 P	Me	Max	*p*
Codominant	AA	21	1.0–2.0	1.5	4.0	0.230191
	AG	98	1.0–2.5	1.5	6.0	
	GG	57	1.0–2.0	1.5	6.5	
Dominant	GG	57	1.0–2.0	1.5	6.5	0.0915
	AG + AA	119	1.0–2.0	1.5	6.0	
Overdominant	AG	98	1.0–2.5	1.5	6.0	0.1340
	AA + GG	78	1.0–2.0	1.5	6.5	
Recessive	AA	21	1.0–2.0	1.5	4.0	0.8892
	AG + GG	155	1.0–2.0	1.5	6.5	
Age at clinical onset (years)						
Model of inheritance	Genotype	*n*	25–75 P	Me	Max	*p*
Codominant	AA	21	24.8–38.5	29.0	62.0	0.297682
	AG	98	24.0–37.0	29.0	60.0	
	GG	57	21.0–34.0	28.0	64.0	
Dominant	GG	57	21.0- 34.0	28.0	64.0	0.1568
	AG + AA	119	24.0–37.8	29.0	62.0	
Overdominant	AG	98	24.0–37.0	29.0	60.0	0.4902
	AA + GG	78	22.0–34.0	29.0	64.0	
Recessive	AA	21	24.8–38.5	29.0	62.0	0.3239
	AG + GG	155	23.0–34.8	29.0	64.0	

*n*—number of individuals, 25–75 P—interquartile ranges, Me—median, Max—maximum, *p*—statistical significance.

**Table 6:** Analysis of the relationship between the rs7385804 *TFR2* polymorphism and selected clinical parameters.

*TFR2* rs7385804						
EDSS 2019 (Points)						
Model of Inheritance	Genotype	*n*	25–75 P	Me	Max	*p*
Codominant	AA	60	1.0–3.3	1.5	6.5	0.602484
	AC	88	1.0–3.0	1.5	6.5	
	CC	28	1.0–2.0	1.5	4.5	
Dominant	AA	60	1.0–3.3	1.5	6.5	0.4650
	AC + CC	116	1.0–2.3	1.5	6.5	
Overdominant	AC	88	1.0–3.0	1.5	6.5	0.9647
	AA + CC	88	1.0–2.5	1.5	6.5	
Recessive	CC	28	1.0–2.0	1.5	4.5	0.3754
	AA + AC	148	1.0–3.0	1.5	6.5	
EDSS at diagnosis (points)						
Model of inheritance	Genotype	*n*	25–75 P	Me	Max	*p*
Codominant	AA	60	1.0–2.0	1.5	6.0	0.802378
	AC	88	1.0–2.0	1.5	6.5	
	CC	28	1.0–2.0	1.5	4.0	
Dominant	AA	60	1.0–2.0	1.5	6.0	0.6100
	AC + CC	116	1.0–2.0	1.5	6.5	
Overdominant	AC	88	1.0–2.0	1.5	6.5	0.9427
	AA + CC	88	1.0–2.0	1.5	6.0	
Recessive	CC	28	1.0–2.0	1.5	4.0	0.5736
	AA + AC	148	1.0–2.0	1.5	6.5	
Age at clinical onset (years)						
Model of inheritance	Genotype	*n*	25–75 P	Me	Max	*p*
Codominant	AA	60	24.0–38.0	29.5	60.0	0.569396
	AC	88	15.0–28.0	28.0	64.0	
	CC	28	15.0–28.0	28.0	62.0	
Dominant	AA	60	24.0–38.0	29.5	60.0	0.3818
	AC + CC	116	22.5–34.0	28.0	64.0	
Overdominant	AC	88	22.0–34.0	28.0	64.0	0.2952
	AA + CC	88	24.0–38.0	29.0	62.0	
Recessive	CC	28	24.5–36.5	28.0	62.0	0.7661
	AA + AC	148	23.0–35.5	29.0	64.0	

*n*—number of individuals, 25–75 P—interquartile ranges, Me—median, Max—maximum, *p*—statistical significance.

**Table 7:** Analysis of the relationship between the rs1049269 *TF* polymorphism and selected clinical parameters.

	TF rs1049269 Codominant Model						
	Genotype						
Clinical Parameters	CC			CT			*p*
	*n*	Me	25–75 P	*n*	Me	25–75 P	
EDSS 2019 (points)	138	1.5	1.0–3.0	38	1.5	1.0–2.0	0.1925
EDSS at diagnosis (points)	138	1.5	1.0–2.0	38	1.0	1.0–1.5	0.0236
Age at clinical onset (years)	138	29.0	23.0–38.0	38	29.0	25.0–34.0	0.6976

*n*—number of individuals, Me—median, 25–75 P—interquartile ranges, *p*—statistical significance.

**Table 8:** Analysis of the relationship between the rs10421768 *HAMP* polymorphism and selected clinical parameters.

*HAMP* rs10421768 (Models od Inheritance)																		
Clinical Parameters		Codominant					Dominant				Overdominant				Recessive			
		AA	AG	GG	X^2^	*p*	AA	AA + GG	X^2^	*p*	GG + AA	AG	X^2^	*p*	GG	AG + AA	X^2^	*p*
Autoimmune diseases	No	95	59	10	1.5	0.4844	95	69	0.3	0.593	105	59	0.9	0.3326	10	154	0.8	0.3798
	Yes	6	6	0			6	6			6	6			0	12		
Family history of autoimmune diseases	No	73	48	7	0.1	0.9566	73	55	0.0	0.8767	80	48	0.1	0.7992	7	121	0.0	0.8424
	Yes	28	17	3			28	20			31	17			3	45		
De novo phenotype	No	39	24	4	0.1	0.9681	39	28	0.0	0.863	43	24	0.1	0.8113	4	63	0.0	0.8972
	Yes	62	41	6			62	47			68	41			6	103		
Relapses	No	67	40	5	1.2	0.5365	67	45	0.7	0.3888	72	40	0.2	0.6589	5	107	0.8	0.3574
	Yes	34	25	5			34	30			39	25			5	59		
MS family history	No	91	59	9	0.0	0.9892	91	68	0.0	0.9	100	59	0.0	0.8833	9	150	0.0	0.9701
	Yes	10	6	1			10	7			11	6			1	16		
Number of occupied systems	One	37	31	5	4.2	0.3736	37	36	3.4	0.1795	42	31	3.5	0.1748	5	68	0.6	0.7339
	Two	46	20	4			46	24			50	20			4	66		
	Three	18	14	1			18	15			19	14			1	32		
MS onset	SF	37	31	5	2.3	0.3151	37	36	2.3	0.1312	42	31	1.6	0.2016	5	68	0.3	0.5743
	MF	64	34	5			64	39			69	34			5	98		
MS disease course	PP	0	1	0	2.2	ne	0	1	1.5	ne	0	1	2.0	ne	0	1	0.3	ne
	RR	99	62	10			99	72			109	62			10	161		
	SP	2	2	0			2	2			2	2			0	4		
MS history in side line	No	94	61	10	0.7	0.6881	94	71	0.2	0.666	104	61	0.0	0.9679	10	155	0.7	0.4018
	Yes	7	4	0			7	4			7	4			0	11		
MS history in straight line	No	98	65	9	4.4	ne	98	74	0.5	ne	107	65	2.4	ne	9	163	2.8	ne
	Yes	3	0	1			3	1			4	0			1	3		

MS—multiple sclerosis, SF—single focal, MF—multi focal, PP—primary progressive multiple sclerosis, RR—relapsing–remitting multiple sclerosis, SP—secondary progressive multiple sclerosis, ne—not estimable, X^2^—Chi-square, *p*—statistical significance.

**Table 9:** Analysis of the relationship between the rs3811647 *TF* polymorphism and selected clinical parameters.

*TF* rs3811647 (Models od Inheritance)																		
Clinical Parameters		Codominant					Dominant				Overdominant				Recessive			
		AA	AG	GG	X^2^	*p*	GG	AG + AA	X^2^	*p*	AA + GG	AG	X^2^	*p*	AA	AG + GG	X^2^	*p*
Autoimmune diseases	No	95	59	10	1.5	0.4844	53	111	0.0	0.9423	71	93	1.0	0.3127	18	146	2.1	0.1491
	Yes	6	6	0			4	8			7	5			3	9		
Family history of autoimmune diseases	No	73	48	7	0.1	0.9566	43	85	0.3	0.5773	58	70	0.2	0.6655	15	113	0.0	0.8871
	Yes	28	17	3			14	43			20	28			6	42		
De novo phenotype	No	39	24	4	0.1	0.9681	23	44	0.2	0.6669	28	39	0.3	0.5978	5	62	2.0	0.1527
	Yes	62	41	6			34	75			50	59			16	93		
Relapses	No	67	40	5	1.2	0.5365	36	76	0.0	0.9274	45	67	2.1	0.1448	9	103	4.4	0.0354
	Yes	34	25	5			21	43			33	31			12	50		
MS family history	No	91	59	9	0.0	0.9892	49	110	1.8	0.1750	69	90	0.6	0.4527	20	139	0.7	0.4195
	Yes	10	6	1			8	9			9	8			1	16		
Number of occupied systems	One	37	31	5	4.2	0.3736	22	51	0.3	0.8618	29	44	1.6	0.4521	7	66	1.6	0.4521
	Two	46	20	4			24	46			35	35			11	59		
	Three	18	14	1			33	22			14	19			3	30		
MS onset	SF	37	31	5	2.3	0.3151	22	51	0.3	0.5924	29	44	1.1	0.3032	7	66	0.6	0.4209
	MF	64	34	5			35	8			49	54			14	89		
MS disease course	PP	0	1	0	2.2	ne	0	1	0.6	ne	0	1	1.4	ne	0	1	0.7	ne
	RR	99	62	10			56	115			77	94			21	150		
	SP	2	2	0			1	3			1	3			0	4		
MS history in side line	No	94	61	10	0.7	0.6881	52	113	0.9	0.3401	72	93	0.5	0.4819	20	145	0.1	0.7647
	Yes	7	4	0			5	6			6	5			1	10		
MS history in straight line	No	98	65	9	4.4	ne	55	117	0.6	ne	76	96	0.1	ne	21	151	0.6	ne
	Yes	3	0	1			2	2			2	2			0	4		

MS—multiple sclerosis, SF—single focal, MF—multi focal, PP—primary progressive multiple sclerosis, RR—relapsing–remitting multiple sclerosis, SP—secondary progressive multiple sclerosis, ne—not estimable, X^2^—Chi-square, *p*—statistical significance.

**Table 10:** Analysis of the relationship between the rs3811647 *TFR2* polymorphism and selected clinical parameters.

*TFR2* rs7385804 (Models od Inheritance)																		
Clinical Parameters		Codominant					Dominant				Overdominant				Recessive			
		AA	AC	CC	X^2^	*p*	AA	AC + CC	X^2^	*p*	AA + CC	AC	X^2^	*p*	AA + CC	CC	X^2^	*p*
Autoimmune diseases	No	54	84	26	1.7	0.4325	54	110	1.4	0.2298	80	84	1.4	0.2329	138	10	0.0	0.9409
	Yes	6	4	2			6	6			8	4			10	2		
Family history of autoimmune diseases	No	47	62	19	1.5	0.4689	47	81	1.4	0.2311	66	62	0.5	0.4996	109	19	0.4	0.5292
	Yes	13	26	9			13	48			22	26			39	9		
De novo phenotype	No	23	35	9	0.5	0.7683	23	44	0.0	0.9586	32	35	0.2	0.6424	58	9	0.5	0.4826
	Yes	37	53	19			37	72			56	53			90	19		
Relapses	No	35	59	18	1.2	0.5554	35	25	1.1	0.2943	53	59	0.9	0.3485	94	18	0.0	0.9381
	Yes	25	29	10			77	39			35	29			54	10		
MS family history	No	56	77	26	1.6	0.4420	56	103	0.9	0.3352	82	77	1.6	0.2033	133	26	0.2	0.6240
	Yes	4	11	2			4	13			6	11			15	2		
Number of occupied systems	One	23	38	12	5.5	0.2367	23	50	1.3	0.5262	35	38	4.7	0.0952	61	12	2.8	0.2492
	Two	23	39	8			23	47			31	39			62	8		
	Three	14	8	8			14	19			22	11			25	8		
MS onset	SF	23	38	12	0.4	0.8304	23	37	0.4	0.5438	35	38	0.2	0.6472	61	12	0.0	0.8720
	MF	37	50	16			50	66			53	50			87	16		
MS disease course	PP	1	0	0	2.9	ne	1	0	2.4	ne	1	0	1.0	ne	1	0	1.0	ne
	RR	57	86	28			57	114			85	86			143	28		
	SP	2	2	0			2	2			2	2			4	0		
MS history in side line	No	57	82	26	0.2	0.8840	57	108	0.2	0.6232	83	82	0.1	0.7562	139	26	0.0	0.8319
	Yes	3	6	2			3	8			5	6			9	2		
MS history in straight line	No	60	84	28	4.1	ne	60	112	2.1	ne	88	84	4.1	ne	144	28	0.8	ne
	Yes	0	4	0			0	4			0	4			4	0		

SF—single focal, MF—multi focal, PP—primary progressive multiple sclerosis, RR—relapsing–remitting multiple sclerosis, SP—secondary progressive multiple sclerosis, ne—not estimable, X^2^—Chi-square, *p*—statistical significance.

**Table 11:** Analysis of the relationship between the rs1049269 *TF* polymorphism and selected clinical parameters.

		*TF* rs1049269 (Models od Inheritance)			
Clinical Parameters		Codominant			
		CC	CT	X^2^	*p*
Autoimmune diseases	No	128	36	0.2	0.6685
	Yes	10	2		
Family history of autoimmune diseases	No	101	27	0.1	0.7941
	Yes	37	11		
De novo phenotype	No	54	13	0.3	0.5813
	Yes	84	25		
Relapses	No	87	25	0.1	0.7560
	Yes	51	13		
MS family history	No	124	35	0.2	0.6784
	Yes	14	3		
Number of occupied systems	One	62	11	3.2	0.1973
	Two	51	19		
	Three	25	8		
MS onset	SF	62	11	3.1	0.0775
	MF	76	27		
MS disease course	PP	1	0	1.4	0.4924
	RR	133	38		
	SP	4	0		
MS history in side line	No	129	36	0.1	0.7772
	Yes	9	2		
MS history in straight line	No	135	37	0.0	0.8673
	Yes	3	1		

SF—single focal, MF—multi focal, PP—primary progressive multiple sclerosis, RR—relapsing–remitting multiple sclerosis, SP—secondary progressive multiple sclerosis, X^2^—Chi-square, *p*—statistical significance.

## Data Availability

Data is available to the readers upon reasonable request.
